# Efficient and Divergent Enantioselective Syntheses of DHPVs and Anti-Inflammatory Effect on IEC-6 Cells

**DOI:** 10.3390/molecules25092215

**Published:** 2020-05-08

**Authors:** Hyun Su Kim, Sungkyun Chung, Moon-Young Song, Changjin Lim, Hyeyoung Shin, Joonseong Hur, Hyuk Kwon, Young-Ger Suh, Eun-Hee Kim, Dongyun Shin, Seok-Ho Kim

**Affiliations:** 1 Department of Pharmacy, College of Pharmacy and Institute of Pharmaceutical Sciences, CHA University, 120 Haeryong-ro, Pocheon 11160, Korea; khs8812@snu.ac.kr (H.S.K.); wjdtjdrbs123@naver.com (S.C.); wso219@naver.com (M.-Y.S.); onam54@gmail.com (H.S.); sed0guitar@naver.com (H.K.); ygsuh@cha.ac.kr (Y.-G.S.); 2 School of Pharmacy, Jeonbuk National University, Jeonju 54896, Korea; limcj@jbnu.ac.kr; 3 Natural Products Research Institute, Korea Institute of Science and Technology (KIST), 679 Saimdang-ro, Gangneung 25451, Korea; hjs1120@kist.re.kr; 4 College of Pharmacy, Gachon University, 191 Hambangmoe-ro, Yeonsu-gu, Incheon 21936, Korea

**Keywords:** divergent synthesis, asymmetric dihydroxylation, DHPV, anti-inflammatory effect, NF-κB, IκBα

## Abstract

Despite numerous reports on the beneficial effects of catechin or epicatechin contained in tea and cacao extract on human health, a conclusive and precise molecular mechanism has not been elucidated. Metabolism of chemical compounds in gut microbiota recently gained significant attention, and extensive studies have been devoted in this field. In conjunction with these results, our group focused on the anti-inflammatory effects of both enantiomers of DHPV (5-(3′,4′-dihydroxyphenyl)-γ-valerolactone), produced in the intestine by microbiota metabolism, on IEC-6 cells. Divergent and efficient enantioselective synthesis of (*S*)- and (*R*)-DHPV was efficiently achieved by cross-metathesis and Sharpless asymmetric dihydroxylation as a key reaction for four steps in 16% and 14% overall yields, respectively. The anti-inflammatory effects of two enantiomers were tested on IEC-6 cells, and we found that (*S*)-DHPV was more active than (*R*)-DHPV. This result implicates that the metabolite produced in the gut has beneficial effects on IEC-6 cells of rat intestines, and the chirality of the metabolite is important for its anti-inflammatory activity. This also provided information for the future discovery of novel small molecular therapeutics for the treatment of inflammatory bowel disease.

## 1. Introduction

Inflammatory bowel disease (IBD) is a chronic and multifactorial inflammatory condition of the gastrointestinal tract, related to an infectious and immunological imbalance of the intestinal mucosa [1,2]. The incidence and prevalence of IBD has relentlessly climbed for past several decades [3], and it is known to be one of the most important risk factors for developing colorectal cancer [4]. IBD is most commonly classified into two principal types: Crohn’s disease (CD) and ulcerative colitis (UC). CD can involve any segment of the gastrointestinal tract and causes non-continuous whole layer inflammation, while UC occurs in the inner lining of the large intestine or rectum and causes continuous inflammation of the colonic mucosa [5,6]. This involves immunity, damage, and patient’s symptoms such as diarrhea and hematochezia [7]. These responses are controlled by nuclear factor kappa-light-chain-enhancer of activated B cells (NF-κB), which are master regulators of the inflammatory reaction. Dysregulation of NF-κB signaling is well known to patients with IBD and are associated with the rapid and acute production of diverse pro-inflammatory mediators [8]. Therefore, pharmacological attempts to block the activation of NF-κB signaling are needed to develop new therapeutic strategies in IBD.

Metabolism in the body plays an important role in the elimination of xenobiotics absorbed by diverse route. Among them, the liver is the major organ contributing to these transformations of phase I and phase II metabolisms [9]. Sometimes, metabolites produced in these biological processes can be newly discovered therapeutics, for example cetirizine or oxazepam. Recently, growing interests in metabolism on the intestine and its effects on brain have also been reported.

**Figure 1:**
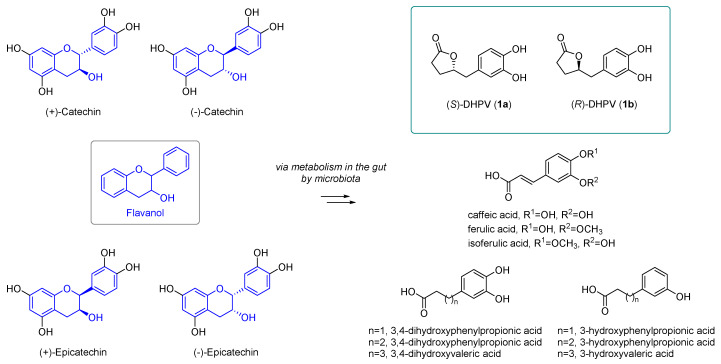
Structures of catechin, epicatechin, and metabolites, including 5-(3′,4′-dihydroxyphenyl)-γ-valerolactones (DHPVs).

Flavanols, such as (−)-catechin, (−)-epicatechin, and each enantiomer, are widely found in red wine, green tea, and cocoa extract (Figure 1). Catechins exist in four isomers, and the enantiomers can be separated by chiral HPLC chromatography [10]. Many research groups have investigated the molecular mechanism, signaling pathway, and metabolisms of polyphenols due to their well-known antioxidant and free radical scavenging activities. However, beneficial effects of these compounds on human health were not conclusive, because these compounds are not readily absorbed into the body as their original structures. Rather, these flavanols undergo metabolism into smaller structures in the gut by microbiota. These metabolites, produced in the intestine, were smoothly absorbed into the bloodstream, even though their potency was decreased compared to the parent molecule.

Catechin and epicatechin undergo intestinal metabolism, producing diverse metabolites including caffeic acid, ferulic acid, isoferulic acid, and so on (Figure 1) [11]. Among the various microbial metabolites from polyphenols in the human body, 5-(3′,4′-dihydroxyphenyl)-γ-valerolactone (DHPV) is the representative metabolite of flavanols containing the intact catechin and epicatechin moiety. DHPV, well-known for its antioxidant activities, such as its protective effect against UVB induced wrinkle formation and neuroprotective effect in the brain, can exist as two enantiomers by the stereocenter in γ-position of butyrolactone moiety. However, their structures are represented in ambiguous or racemic forms in numerous literatures, and we became interested in confirming which DHPV enantiomer exhibited more potent biological activities, because there are numerous reports on the completely different biological activities of enantiomers [12,13].

Herein, we reported the enantioselective synthesis of (*S*)- and (*R*)-DHPV by the divergent and efficient approach. We also evaluated the effects of both enantiomers on lipopolysaccharide (LPS) induced phosphorylation and degradation of inhibitor of κB alpha (IκBα), the major key regulators of NF-κB signaling pathway, to test the mechanism of anti-inflammatory effects on IEC-6 cells.

## 2. Results and Discussion

### 2.1. Retrosynthetic Analysis of 5-(3′,4′-Dihydroxyphenyl)-γ-Valerolactone (DHPV)

Until now, four groups completed the racemic or enantioselective total synthesis of DHPV. The Watanabe [14] group reported the first racemic synthesis of DHPV from benzyl epoxide as a key intermediate in a four step synthesis, and the Lambert [15] group also reported the racemic synthesis of DHPV via Heck reaction and dehydrative lactonization for a total of eight synthetic steps. The first enantioselective synthesis of DHPV was completed by the Nakajima [16,17] group from optically pure epoxide in seven steps, and the Curti [18] group achieved the relatively recent asymmetric synthesis of DHPV via the concise and catalytic enantioselective Mukaiyama aldol reaction in six steps. All of the previous reports of DHPV were accomplished in four to eight steps, but the only racemic DHPV was obtained through the minimal synthetic pathway.

**Scheme 1:**
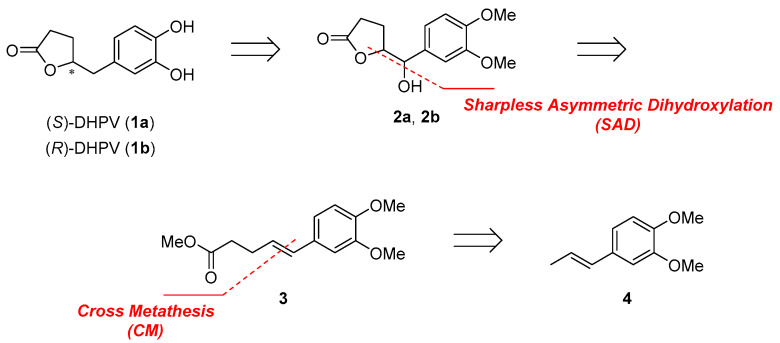
Retrosynthetic analysis of DHPV.

Our group planned the divergent synthesis and biological evaluation of both enantiomers. Representative retrosynthetic analysis of DHPV was summarized in Scheme 1. (*S*) or (*R*)-DHPV could be obtained by deoxygenation of benzylic alcohol and demethylation from **2a** or **2b** [19,20]. δ-Hydroxy-γ-valerolactone (**2a**, **2b**) could be formed by Sharpless Asymmetric Dihydroxylation (SAD) [21] and spontaneous lactonization from common intermediate alkene **3**. Alkene **3** can be obtained from **4** by cross metathesis, and styrene **4** was used to lower homo-dimerization of terminal olefin [22]. Although we planned the synthesis of γ,δ-unsaturated ester **3** by Johnson–Claisen rearrangement [15] instead of cross metathesis at the beginning of this project, we couldn’t obtain meaningful result.

### 2.2. Divergent Synthesis of DHPV

**Scheme 2:**
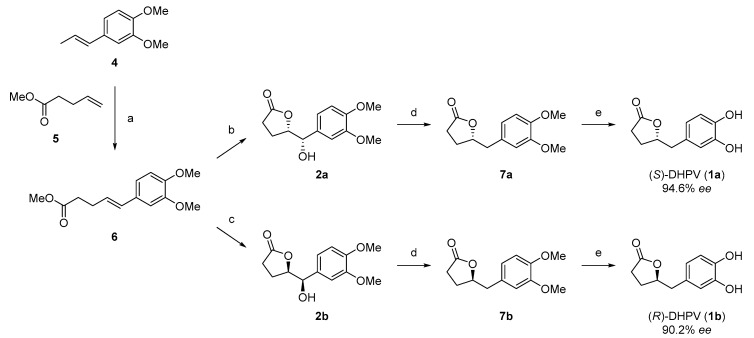
Divergent and enantioselective synthesis of DHPV. (**a**) **4**, **5**, Grubbs 2nd generation catalyst, CH_2_Cl_2_, reflux, 59% (70% BORSM); (**b**) AD-mix-α, MeSO_2_NH_2_, *t*-BuOH/H_2_O (1/1), 62%; (**c**) AD-mix-β, MeSO_2_NH_2_, *t*-BuOH/H_2_O (1/1), 55%; (**d**) Pd(OH)_2_, H_2_, MeOH, r.t. 72% (**7a**) and 68% (**7b**); and (**e**) BBr_3_, CH_2_Cl_2_, 0 °C to r.t., 60% (**1a**) and 62% (**1b**).

Synthesis of (*S*)- and (*R*)-DHPV began with construction of the phenylpentanoate scaffold (Scheme 2). Cross metathesis of commercially available (*E*)-3,4-dimethoxyphenylpropene (**4**) and methyl 4-pentenoate (**5**) by Grubbs 2nd generation catalyst afforded the desired product **6** in 59% yield. Interestingly, only (*E*)-olefinic isomer was obtained, which was identified through ^1^H-NMR analysis (Supplementary Materials). One pot Sharpless asymmetric dihydroxylation (AD-mix-α and AD-mix-β, respectively) and concomitant lactonization of alkene **6** provided **2a** and **2b** in 62% and 55% for each isomer, respectively. Then, alcohols **2a** and **2b** were subjected to hydrogenolysis, providing **7a** and **7b** by Pearlman’s catalyst in good yields of 72% and 68%, which were transformed to **1a** and **1b** by demethylation with BBr_3_ in 60% and 62%, respectively. The ^1^H- and ^13^C-NMR spectral data of both enantiomers were compared and matched well. A detailed summary of the spectrum was represented in Supplementary Materials.

The enantiomeric purities of asymmetric dihydroxylation products, **2a** and **2b**, and final (*S*)- and (*R*)-DHPV, **1a** and **1b**, were confirmed by the analytical chiral HPLC experiment (Supplementary Materials), according to previous report of Suh group [23]. The synthetic final enantiomers were pure enough for further biological study, with the enantioselectivity of 97.3:2.7 for **1a** and 95.1:4.9 for **1b**.

### 2.3. Anti-Inflammatory Activity of (R)- and (S)-DHPV on IEC-6 Cells

NF-κB is a major regulator of the immune system, and the activation of NF-κB induces inflammation and carcinogenesis [6,8,24]. Dysregulated immune responses were implicated in the pathogenesis of IBD, and the transcription factor, NF-κB, was found to be one of the key regulatory components in this complex scenario [25]. It is well known that the expression and activation of NF-κB was strongly induced in the inflamed gut of IBD patients. In particular, macrophages and epithelial cells isolated from inflamed gut specimens from IBD patients showed increased levels of the NF-κB subunit [26]. Interestingly, the amount of activated NF-κB significantly correlated with the severity of intestinal inflammation [27]. Classic activation of NF-κB can be initiated by various stimuli, including bacterial cell wall components like lipopolysaccharide (LPS), pro-inflammatory cytokines, viruses, and DNA damaging agents. These triggering substances stimulate a phosphorylation of Iκβs, and the translocation of NF-κB into the nucleus promotes the transcription of genes that encode inflammation-related proteins [28,29]. Therefore, the measurement of phosphorylation and degradation of IκBα could be the simple but valid assay for the anti-inflammatory ability of synthesized compounds through the suppression of NF-κB in IBD.

**Figure 2:**
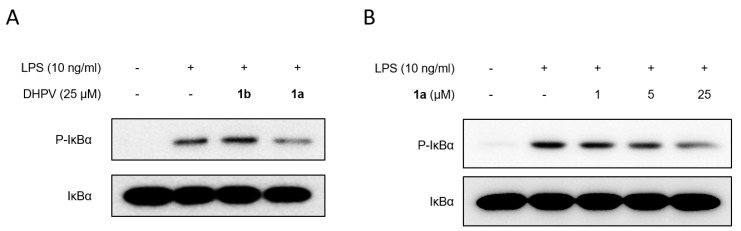
Biological activity of synthesized (*R*)- and (*S*)-DHPV. (**A**) The effects of **1b** (*R*)-DHPV (25 μM) and **1a** (*S*)-DHPV (25 μM) on the phosphorylation and degradation of IκBα were determined by Western blot analysis in IEC-6 cells exposed to lipopolysaccharides (LPS; 10 ng/mL) for 1 h. (**B**) The effect of **1a** (*S*)-DHPV on the expression of phosphorylation and degradation of IκBα was analyzed dose dependently by Western blot analysis in LPS (10 ng/mL)-treated IEC-6 cells.

To evaluate the preventive effects of DHPVs against inflammatory responses, we pretreated rat intestinal epithelial IEC-6 cells with each DHPV (25 μM) for 1 h prior to the LPS stimulation (Figure 2A). The inhibitory effect of (*R*)-DHPV (**1b**) and (*S*)-DHPV (**1a**) on phosphorylation and degradation of IκBα expression were tested under the same conditions for comparison. We observed that the inhibitory effect of (*S*)-DHPV on the phosphorylation and degradation of IκBα was more significant than that of (*R*)-DHPV in LPS-treated IEC-6 cells (Figure 2A). Comparing the anti-inflammatory effects of (*S*)-DHPV with (*R*)-DHPV, in terms of suppression of phosphorylation and degradation of IκBα, (*S*)-DHPV was found to be more effective in suppressing NF-κB signaling than (*R*)-DHPV. We also observed that (*S*)-DHPV (**1a**) downregulated LPS-induced phosphorylation and degradation of IκBα in a dose dependent manner (Figure 2B). These results indicated that (*S*)-DHPV can protect against LPS-induced inflammation by inhibiting NF-κB activation through repression of IκBα degradation.

## 3. Materials and Methods

### 3.1. General Information

#### 3.1.1. General Methods and Materials

Unless otherwise noted, all the reactions were run under nitrogen (N_2_) atmosphere in anhydrous condition. DHPVs were dissolved in dimethyl sulfoxide (DMSO) as stock solution and stored at −20 °C. DMSO was purchased from Sigma Chemical Co. (St. Louis, MO, USA). Lipopolysaccharide (LPS) was purchased from Sigma Chemical Co. (St. Louis, MO, USA). Antibodies for Phosphorylated-IkBα and total IkBα were purchased from Cell Signaling Technology (Danvers, MA, USA).

#### 3.1.2. Materials

All reagents were obtained from Sigma-Aldrich (St. Louis, MO, USA) or TCI (Tokyo, Japan), and solvents were purchased from Daejung (Siheung, Korea) and used without further purification. Dichloromethane (CH_2_Cl_2_) was distilled from calcium hydride (CaH_2_). Methanol (MeOH) was distilled from magnesium sulfate (MgSO_4_).

#### 3.1.3. Instrumentation

^1^H and ^13^C-spectra were recorded on a 500 MHz JNM-ECZ 500R (JEOL, Tokyo, Japan). Chemical shifts are recorded as a δ value (δ 7.26 for ^1^H and δ 77.0 for ^13^C). Data were represented as follows: chemical shift, multiplicity (s = singlet, d = doublet, t = triplet, q = quartet, m = multiplet, and br = broad), integration, coupling constant in Hz, and assignment. Infrared (IR) spectra were measured on a 1600 FT–IR spectrometer (Perkin-Elmer, Waltham, MA, USA). High resolution mass spectrometry data were recorded by JMS-700 (JEOL, Tokyo, Japan) (FAB or ESI), and values were calculated to four decimal places from the molecular formula. Melting point (m.p.) was obtained using MEL-TEMP^®^ (Cole-Parmer, Staffordshire, UK).

### 3.2. Experimental Part

*(E)-Ethyl-5-(3,4-dimethoxyphenyl)pent-4-enoate* (**6**). To a stirred solution of ethyl pent-4-enoate (967mg, 7.54 mmol) and 4-(1-propenyl)-1,2-dimethoxybenzene (2.67 g, 15.1 mmol, 2.0 equiv.) in CH_2_Cl_2_ (50 mL) was added Grubbs 2nd generation catalyst (310 mg, 0.38 mmol, 0.05 equiv.) and then heated to reflux for 6 h. Then, the reaction mixture was cooled to room temperature, and solvent was removed under reduced pressure. EtOAc/*n*-hexane (1:10, 30 mL) was added, and the solid was filtered to provide the 1.76 g dimer of styrene. The filtrate was purified by silica gel column chromatography to afford **6** (1.17 g, 4.44 mmol), a colorless oil with recovered ethyl pent-4-enoate (160 mg); Yield 59% (70% BORSM); ^1^H-NMR (500 MHz, CDCl_3_) δ 6.88 (d, *J* = 1.7 Hz, 1H), 6.85 (dd, *J* = 8.0, 1.7 Hz, 1H), 6.78 (d, *J* = 8.5 Hz, 1H), 6.35 (d, *J* = 15.5 Hz, 1H), 6.06 (dt, *J* = 16.0, 6.6 Hz, 1H), 4.13 (q, *J* = 7.3 Hz, 2H), 3.88 (s, 3H), 3.86 (s, 3H), 2.52–2.44 (m, 4H), and 1.24 (t, *J* = 7.2 Hz, 3H); ^13^C-NMR (125 MHz, CDCl_3_) δ 173.1, 149.0, 148.5, 130.6, 130.6, 126.6, 119.1, 111.1, 108.6, 60.4, 55.9, 55.8, 34.2, 28.3, and 14.3; FT-IR (thin film, neat) ν_max_ 2980, 2358, 2341, 2040, 1732, 1514, 1263, 1024, and 966 cm^−1^; and HRMS (ESI+) found 265.1444 (calculated for C_15_H_21_O_4_ ([M + H]^+^): 265.1434).

*(S)-5-((S)-(3,4-dimethoxyphenyl)(hydroxy)methyl)dihydrofuran-2(3H)-one* (**2a**). To a stirred solution of AD-mix-α (1.47 g, 1.40g/mmol) and methane sulfonamide (120.9 mg, 1.27 mmol, 1.21 equiv.) in *t*-BuOH/H_2_O (1:1, 30 mL) was added **6** (280 mg, 1.05 mmol) in *t*-BuOH (10 mL) and stirred at 0 °C for 72 h. Then, EtOAc (100 mL) was added to the reaction mixture and the organic layer was separated. The aqueous layer was extracted again with EtOAc (two times, 50 mL) and the combined organic layer was washed with H_2_O (two times, 10 mL). Then, the organic layer was dried over MgSO_4_ and the solvent was removed under reduced pressure. The lactone was purified by silica gel column chromatography (EtOAc/*n*-hexane, 2:1) to afford **2a** (164 mg, 0.65 mmol) as a white solid; Yield 62%; m.p. 138–140 °C; ^1^H-NMR (500 MHz, CDCl_3_) δ 6.91 (d, *J* = 1.7 Hz, 1H), 6.89 (dd, *J* = 8.0, 1.7 Hz, 1H), 6.83 (d, *J* = 8.0 Hz, 1H), 4.63–4.59 (m, 2H), 3.87 (s, 3H), 3.86 (s, 3H), 2.50–2.43 (m, 3H), and 2.03–1.95 (m, 2H); ^13^C-NMR (125 MHz, CDCl_3_) δ 177.0, 149.3, 149.2, 130.8, 119.4, 111.0, 109.7, 83.5, 76.3, 56.0, 55.9, 28.5, and 24.0; FT–IR (thin film, neat) ν_max_ 3477, 2939, 1770, 1514, 1263, and 1095 cm^−1^; and HRMS (ESI+) found 252.0979 (calculated for C_13_H_16_O_5_ ([M]^+^): 252.0992).

*(R)-5-((R)-(3,4-dimethoxyphenyl)(hydroxy)methyl)dihydrofuran-2(3H)-one* (**2b**). This reaction was conducted with AD-mix-β, instead of AD-mix-α. Yield 55%, white solid; m.p. 129–131 °C; ^1^H-NMR (500 MHz, CDCl_3_) δ 6.93 (d, *J* = 2.3 Hz, 1H), 6.91 (dd, *J* = 8.6, 1.7 Hz, 1H), 6.85 (d, *J* = 8.6 Hz, 1H), 4.70–4.62 (m, 2H), 3.88 (s, 3H), 3.87 (s, 3H), 2.75 (bs, 1H), 2.49–2.43 (m, 2H), and 2.03–1.99 (m, 2H); ^13^C-NMR (125 MHz, CDCl_3_) δ 177.2, 149.2, 149.2, 130.9, 119.4, 111.0, 109.8, 83.6, 76.2, 56.0, 55.9, 28.5, and 24.0; FT–IR (thin film, neat) ν_max_ 3477, 2939, 1770, 1516, 1263, 1184, and 1141 cm^−1^; HRMS (ESI+) found 252.1003 (calculated for C_13_H_16_O_5_ ([M]^+^): 252.0992).

*(S)-5-(3,4-dimethoxybenzyl)dihydrofuran-2(3H)-one* (**7a**). To a stirred solution of **2a** (150 mg, 0.603 mmol) in MeOH (20 mL) and N_2_ atmosphere was added Pd(OH)_2_ on activated charcoal (20 mg). Then, nitrogen gas was removed under reduced pressure, and the reaction mixture was charged with a hydrogen gas balloon. After 6 h, the reaction mixture was filtered through a Celite pad, and solvent was removed under reduced pressure. The crude product was purified by silica gel column chromatography (EtOAc/*n*-hexane, 1:1) to afford **2a** (102 mg, 0.43 mmol), a colorless oil; Yield 72%; ^1^H-NMR (500 MHz, CDCl_3_) δ 6.79 (d, *J* = 8.1 Hz, 1H), 6.75–6.71 (m, 2H), 4.71 (quintet, *J* = 6.4 Hz, 1H), 3.85 (s, 3H), 3.84 (s, 3H), 2.96 (dd, *J* = 14.4, 5.8 Hz, 1H), 2.89 (dd, *J* = 14.3, 6.3 Hz, 1H), 2.50–2.22 (m, 3H), and 1.96–1.89 (m, 1H); ^13^C-NMR (125 MHz, CDCl_3_) δ 177.2, 149.0, 148.1, 128.3, 121.6, 112.6, 111.4, 111.3, 80.9, 55.9, 55.9, 28.7, and 27.0; FT-IR (thin film, neat) ν_max_ 3522, 2937, 1770, 1734, 1514, 1261, 1178, 1157, 1141, and 1026 cm^−1^; HRMS (ESI+) found 237.1129 (calculated for C_13_H_17_O_4_ ([M + H]^+^): 237.1121).

*(R)-5-(3,4-dimethoxybenzyl)dihydrofuran-2(3H)-one* (**7b**). Yield 68%, colorless oil; ^1^H-NMR (500 MHz, CDCl_3_) δ 6.80 (d, *J* = 8.0 Hz, 1H), 6.76–6.73 (m, 2H), 4.71 (quintet, *J* = 6.9 Hz, 1H), 3.86 (s, 3H), 3.85 (s, 3H), 2.97 (dd, *J* = 14.3, 5.8 Hz, 1H), 2.89 (dd, *J* = 14.4, 5.8 Hz, 1H), 2.47–2.23 (m, 3H), and 1.97–1.89 (m, 1H); ^13^C-NMR (125 MHz, CDCl_3_) δ 177.2, 149.0, 148.2, 128.4, 121.6, 112.7, 113.4, 80.9, 56.0, 55.9, 40.9, 28.7, and 27.0; FT–IR (thin film, neat) ν_max_ 3477, 2939, 1772, 1516, 1263, 1184, 1143, and 1026 cm^−1^; HRMS (ESI+) found 237.1125 (calculated for C_13_H_17_O_4_ ([M + H]^+^): 237.1121).

*(S)-5-(3,4-dihydroxybenzyl)dihydrofuran-2(3H)-one* (**1a**). To a stirred solution of **7a** (50 mg, 0.212 mmol) in CH_2_Cl_2_ (10 mL) was added to BBr_3_ (100 µL, 1.06 mmol, 5.0 equiv.) at 0 °C, and the reaction mixture was warmed to room temperature. Then, the reaction mixture was quenched with H_2_O (10 mL) at 0 °C, and the organic layer was separated. The aqueous layer was extracted again with CH_2_Cl_2_ (two times, 50 mL), and the combined organic layer was washed with H_2_O (two times, 10 mL). Then, the organic layer was dried over MgSO_4_, and the solvent was removed under reduced pressure. The lactone was purified by silica gel column chromatography (CH_2_Cl_2_/MeOH = 10:1) to afford **1a** (26 mg, 0.127 mmol), a white solid. Yield 60%; m.p. 163–165 °C; ^1^H-NMR (500 MHz, DMSO-*d_6_*) δ 8.74 (bs, 2H), 6.60 (d, *J* = 8.0 Hz, 1H), 6.58 (d, *J* = 2.3 Hz, 1H), 6.44 (dd, *J* = 8.0, 2.3 Hz, 1H), 4.58 (quintet, *J* = 6.7 Hz, 1H), 2.73 (dd, *J* = 13.8, 6.9 Hz, 1H), 2.66 (dd, *J* = 13.7, 5.8 Hz, 1H), 2.41 (dd, *J* = 17.8, 8.6 Hz, 1H), 2.31 (ddd, *J* = 17.8, 9.8, 5.2 Hz, 1H), 2.14–2.07 (m, 1H), and 1.83–1.76 (m, 1H); ^13^C-NMR (125 MHz, DMSO-*d_6_*) δ 177.6, 145.5, 144.4, 128.0, 120.5, 117.2, 115.9, 81.3, 40.2, 28.7, and 27.1; FT–IR (thin film, neat) ν_max_ 3404, 1575, 1517, 1273, and 1186 cm^−1^; HRMS (ESI+) found 209.0811 [calculated for C_11_H_13_O_4_ ([M + H]^+^): 209.0808].

*(R)-5-(3,4-dihydroxybenzyl)dihydrofuran-2(3H)-one* (**1b**). Yield 62%, white solid; m.p. 154−156 °C; ^1^H-NMR (500 MHz, DMSO-*d_6_*) δ 8.78 (bs, 2H), 6.60 (d, *J* = 7.5 Hz, 1H), 6.57 (d, *J* = 1.7 Hz, 1H), 6.44 (dd, *J* = 8.0, 2.3 Hz, 1H), 4.58 (quintet, *J* = 6.7 Hz, 1H), 2.73 (dd, *J* = 14.3, 6.8 Hz, 1H), 2.65 (dd, *J* = 14.3, 6.5 Hz, 1H), 2.41 (dd, *J* = 17.8, 9.2 Hz, 1H), 2.31 (ddd, *J* = 17.2, 9.2, 4.6 Hz, 1H), 2.13–2.08 (m, 1H), and 1.83–1.75 (m, 1H); ^13^C-NMR (125 MHz, DMSO-*d_6_*) δ 177.6, 145.6, 144.4, 127.9, 120.5, 117.2, 115.9, 81.3, 40.2, 28.7, and 27.1; FT-IR (thin film, neat) ν_max_ 3331, 3055, 1762, 1517, 1444, 1265, 1180, and 736 cm^−1^; HRMS (ESI+) found 209.0808 (calculated for C_11_H_13_O_4_ ([M + H]^+^): 209.0808).

### 3.3. Cell Culture and Treatments

Rat intestinal epithelial IEC-6 cells were obtained from the American Type Culture Collection (ATCC, Rockville, MD, USA) and maintained according to the ATCC’s instructions. IEC-6 cells were grown as a monolayer in Dulbecco’s modified Eagle’s medium (HyClone, GE Healthcare, Logan, UT, USA), containing 10% (*v/v*) fetal bovine serum (ATCC), 100 U/mL penicillin, and 100 μg/mL streptomycin and maintained at 37 °C in a humidified atmosphere containing 5% CO_2_. The cells were treated with or without LPS for 1 h, in the presence or absence of DHPV.

### 3.4. Cell Lysates and Western Blot Analysis

Cell monolayers were washed twice with PBS, scraped on ice into cold cell lysis buffer containing protease inhibitor (Roche Applied Science, Mannheim, Germany), and centrifuged to remove the pellet and debris. Western blot analysis was performed as previously described [1]. Proteins were separated by SDS-PAGE and transferred to polyvinylidene fluoride membranes, which were incubated with the primary antibodies, washed, incubated with peroxidase conjugated secondary antibodies, rewashed, and then visualized using an enhanced chemiluminescence system (Thermo Fisher Scientific, Waltham, MA, USA).

## 4. Conclusions

Our results, focused on the metabolite produced by metabolism of the catechins in the intestine and subsequent effects on the intestine, has been disclosed here. In this context, we synthesized both enantiomers of DHPV via the efficient and divergent approach and confirmed their biological activity. The synthesis was satisfactorily achieved with overall yield of 14% to 16% and the enantioselectivity of 97.3:2.7 (**1a**) and 95.1:4.9 (**1b**), respectively. The main cause of the IBD was related to inflammation on the intestine. Therefore, the anti-inflammatory effects of these compounds on rat intestinal epithelial IEC-6 cells were examined. (*S*)-DHPV proved to more efficiently inhibit inflammation induced by LPS by inhibiting the phosphorylation of IκBα, dose-dependently.

Our findings indicate that there was a new rationale to use (*S*)-DHPV as a new therapeutic agent for the prevention and treatment of IBD. Due to the complex mucosal immune system and very broad panel of genes controlled by NF-κB, which contribute to the pathogenesis of IBD, it is necessary to carefully explore the further effects of (*S*)-DHPV on NF-κB signaling.

## Supplementary Materials

The following are available online: ^1^H and ^13^C-NMR spectra and the chiral HPLC analysis.

## References

[1] Kaser A., Lee A.-H., Franke A., Glickman J.N., Zeissig S., Tilg H., Nieuwenhuis E.E., Higgins D.E., Schreiber S., Glimcher L.H. (2008). XBP1 links ER stress to intestinal inflammation and confers genetic risk for human inflammatory bowel disease. Cell.

[2] Halpin S.J., Ford A.C. (2012). Prevalence of symptoms meeting criteria for irritable bowel syndrome in inflammatory bowel disease: Systematic review and meta-analysis. Am. J. Gastroenterol..

[3] Molodecky N.A., Soon S., Rabi D.M., Ghali W.A., Ferris M., Chernoff G., Benchimol E.I., Panaccione R., Ghosh S., Barkema H.W. (2012). Increasing incidence and prevalence of the inflammatory bowel diseases with time, based on systematic review. Gastroenterology.

[4] Kim E.R., Chang D.K. (2014). Colorectal cancer in inflammatory bowel disease: The risk, pathogenesis, prevention and diagnosis. World J. Gastroenterol..

[5] Baumgart D.C., Sandborn W.J. (2012). Crohn’s disease. Lancet.

[6] Roda G., Narula N., Pinotti R., Skamnelos A., Katsanos K., Ungaro R., Burisch J., Torres J., Colombel J.F. (2017). Systematic review with meta-analysis: Proximal disease extension in limited ulcerative colitis. Aliment. Pharm. Ther..

[7] Neish A.S. (2009). Microbes in gastrointestinal health and disease. Gastroenterology.

[8] McDaniel D.K., Eden K., Ringel V.M., Allen I.C. (2016). Emerging roles for noncanonical NF-κB signaling in the modulation of inflammatory bowel disease pathobiology. Inflamm. Bowel Dis..

[9] Liu X., Wang H., Liang X., Roberts M. (2017). Hepatic Metabolism in Liver Health and Disease. Liver Pathophysiology.

[10] Rinaldo D., Batista Jr J.M., Rodrigues J., Benfatti A.C., Rodrigues C.M., Dos Santos L.C., Furlan M., Vilegas W. (2010). Determination of catechin diastereomers from the leaves of Byrsonima species using chiral HPLC-PAD-CD. Chirality.

[11] Monagas M., Urpi-Sarda M., Sanchez-Patan F., Llorach R., Garrido I., Gomez-Cordoves C., Andres-Lacueva C., Bartolome B. (2010). Insights into the metabolism and microbial biotransformation of dietary flavan-3-ols and the bioactivity of their metabolites. Food Funct..

[12] H Brooks W., C Guida W., G Daniel K. (2011). The significance of chirality in drug design and development. Curr. Top. Med. Chem..

[13] Saha D., Kharbanda A., Yan W., Lakkaniga N.R., Frett B., Li H.-Y. (2020). The Exploration of Chirality for Improved Druggability within the Human Kinome. J. Med. Chem..

[14] Watanabe H. (1959). The Chemical Structure of the Intermediate Metabolites of Catechin I–IV: Chemical Properties of the Intermediate Metabolites (G and H) and their Derivatives Oxidative Decomposition of the Intermediate Metabolites (G and H) Synthesis of the Intermediate Metabolites (G and H) Structure of the Intermediate Metabolite (F). J. Agric. Chem. Soc. Jpn..

[15] Lambert J.D., Rice J.E., Hong J., Hou Z., Yang C.S. (2005). Synthesis and biological activity of the tea catechin metabolites, M4 and M6 and their methoxy-derivatives. Bioorg. Med. Chem. Lett..

[16] Nakano S., Hamada M., Kishimoto T., Nakajima N. (2008). Synthesis of γ-valerolactones as the tea catechin metabolites. Heterocycles.

[17] Hamada M., Furuno A., Nakano S., Kishimoto T., Nakajima N. (2010). Synthesis of optically pure lactone metabolites of tea catechins. Synthesis.

[18] Curti C., Brindani N., Battistini L., Sartori A., Pelosi G., Mena P., Brighenti F., Zanardi F., Del Rio D. (2015). Catalytic, Enantioselective Vinylogous Mukaiyama Aldol Reaction of Furan-Based Dienoxy Silanes: A Chemodivergent Approach to γ-Valerolactone Flavan-3-ol Metabolites and δ-Lactone Analogues. Adv. Synth. Catal..

[19] Wu B., Gao X., Yan Z., Chen M.-W., Zhou Y.-G. (2015). C–H Oxidation/Michael Addition/Cyclization Cascade for Enantioselective Synthesis of Functionalized 2-Amino-4*H*-chromenes. Org. Lett..

[20] Shopsowitz K.E., Edwards D., Gallant A.J., MacLachlan M.J. (2009). Highly substituted Schiff base macrocycles via hexasubstituted benzene: A convenient double Duff formylation of catechol derivatives. Tetrahedron.

[21] Kolb H.C., VanNieuwenhze M.S., Sharpless K.B. (1994). Catalytic asymmetric dihydroxylation. Chem. Rev..

[22] Chatterjee A.K., Choi T.-L., Sanders D.P., Grubbs R.H. (2003). A general model for selectivity in olefin cross metathesis. J. Am. Chem. Soc..

[23] Hur J., Kim A.-R., Kim H.S., Lim C., Kim T., Kim T.-A., Sim J., Suh Y.-G. (2020). Concise Synthesis of Catechin Metabolites 5-(3′,4′-Dihydroxyphenyl)-γ-valerolactones (DHPV) in Optically Pure Form and Their Stereochemical Effects on Skin Wrinkle-Reducing Activities. Molecules.

[24] Neish A.S. (2002). The gut microflora and intestinal epithelial cells: A continuing dialogue. Microb. Infect..

[25] Atreya I., Atreya R., Neurath M.F. (2008). NF-κB in inflammatory bowel disease. J. Intern. Med..

[26] Neurath M.F., Pettersson S., Meyer zum Büschenfelde K.H., Strober W. (1996). Local administration of antisense phosphorothiate olignucleotides to the p65 subunit of NF–κB abrogates. Nat. Med..

[27] Rogler G., Brand K., Vogl D., Page S., Hofmeister R., Andus T., Knuechel R., Baeuerle P.A., Schölmerich J., Gross V. (1998). Nuclear factor κB is activated in macrophages and epithelial cells of inflamed intestinal mucosa. Gastroenterology.

[28] Harikumar K.B., Kunnumakkara A.B., Ahn K.S., Anand P., Krishnan S., Guha S., Aggarwal B.B. (2009). Modification of the cysteine residues in IκBα kinase and NF-κB (p65) by xanthohumol leads to suppression of NF-κB–regulated gene products and potentiation of apoptosis in leukemia cells. Blood.

[29] Escobar J., Pereda J., Arduini A., Sandoval J., Sabater L., Aparisi L., López-Rodas G., Sastre J. (2009). Cross-talk between oxidative stress and pro-inflammatory cytokines in acute pancreatitis: A key role for protein phosphatases. Curr. Pharm. Des..

