# Supplementary material for: Efficient and Divergent Enantioselective Syntheses of DHPVs and Anti-Inflammatory Effect on IEC-6 Cells
Source: Molecules. 2020 May 8;25(9):2215. doi: 10.3390/molecules25092215 (PMC7248962; doi:10.3390/molecules25092215)
Supplement: Supplementary file 1 [file molecules-25-02215-s001.pdf]

Supporting Information

Efficient and Divergent Enantioselective Syntheses of DHPVs and Anti-Inflammatory Effect on IEC-6 cells

Hyun Su Kim [1,†], Sungkyun Chung [1,†], Moon-Young Song [1,†], Changjin Lim [2], Hyeyoung Shin [1], Joonseong Hur [3], Hyuk Kwon [1], Young-Ger Suh [1], Eun-Hee Kim [1,*], Dongyun Shin [4,*] and Seok-Ho Kim [1,*]

[1] Department of Pharmacy, College of Pharmacy and Institute of Pharmaceutical Sciences, CHA University, 120 Haeryong-ro, Pocheon 11160, Gyeonggi-do, Korea; khs8812@snu.ac.kr (H. S. Kim), wjdtjdrbs123@naver.com (S. Chung), wso219@naver.com (M. Song), sed0guitar@naver.com (H. Kwon), ygsuh@cha.ac.kr(Y.-G. Suh)

[2] School of Pharmacy, Jeonbuk National University, Jeonju 54896, Republic of Korea; limcj@jbnu.ac.kr (C. Lim)

[3] Natural Products Research Institute, Korea Institute of Science and Technology (KIST), 679 Saimdang-ro, Gangneung 25451, Republic of Korea; hjs1120@kist.re.kr

[4] College of Pharmacy, Gachon University, 191 Hambangmoe-ro, Yeonsu-gu, Incheon 21936, South Korea. Gyeonggi-do, Korea

[†]These authors contributed equally to this work.

Correspondence to: S.-H. Kim (ksh3410@cha.ac.kr), E.-H. K (ehkim@cha.ac.kr) and D. Y. Shin (dyshin@gachon.ac.kr)

Table of Contents

Copies of ^{1}H NMR and ^{13}C NMR Spectra -- S1

Table 1. Comparison of ^{1}H-NMR data for Synthetic and reported DHPV ------------------------------- S8

Chiral HPLC analysis of Synthetic compounds -- S9

^{1}H and ^{13}C NMR Spectra

(*E*)-Methyl 5-(3,4-dimethoxyphenyl)pent-4-enoate (**6**)

^{1}H NMR (CDCl$_3$, 500 MHz)

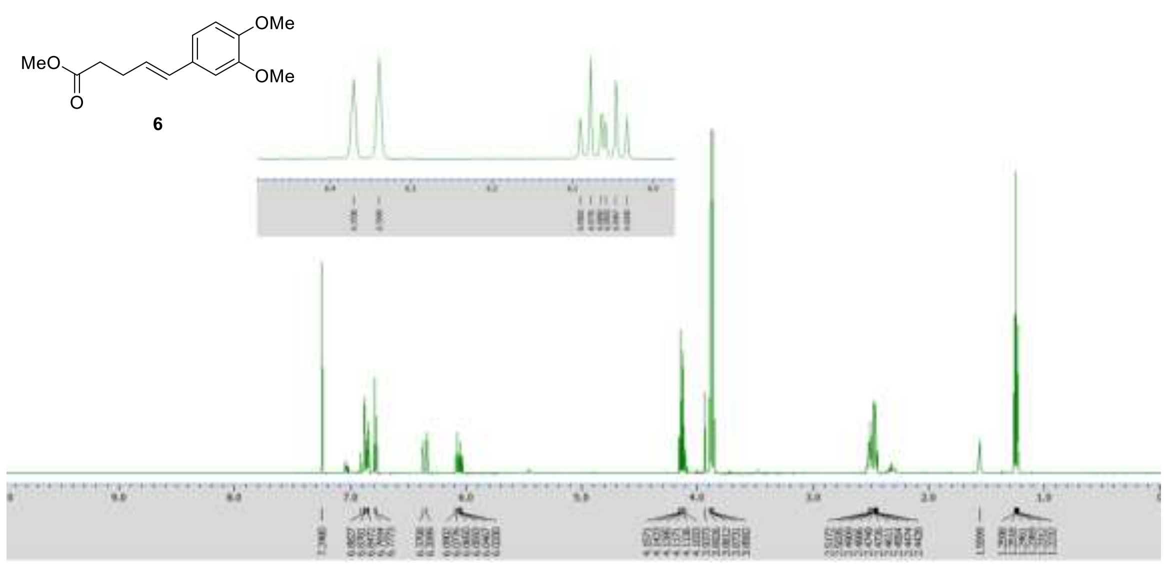

^{13}C NMR (CDCl$_3$, 125 MHz)

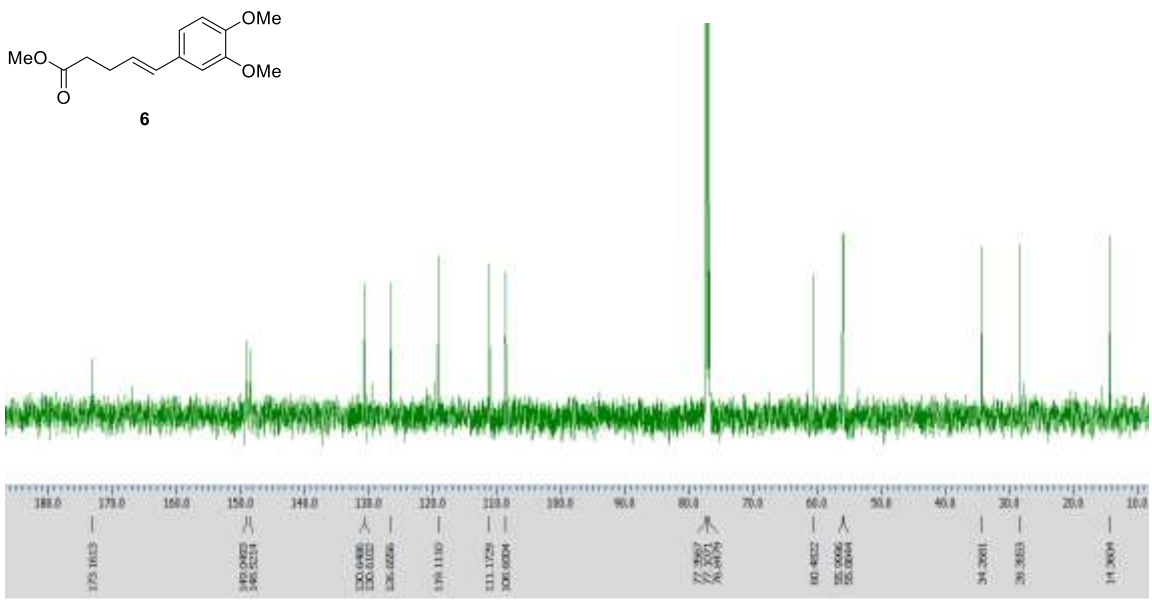

(*S*)-5-((*S*)-(3,4-dimethoxyphenyl)(hydroxy)methyl)dihydrofuran-2(3*H*)-one (**2a**)

^{1}H NMR ($CDCl_3$, 500 MHz)

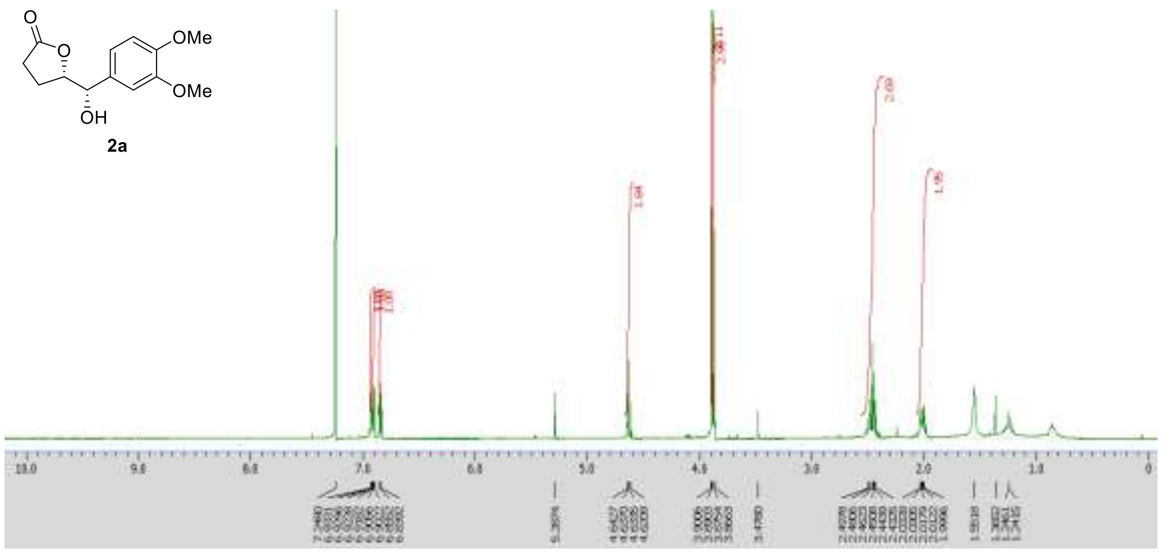

^{13}C NMR ($CDCl_3$, 125 MHz)

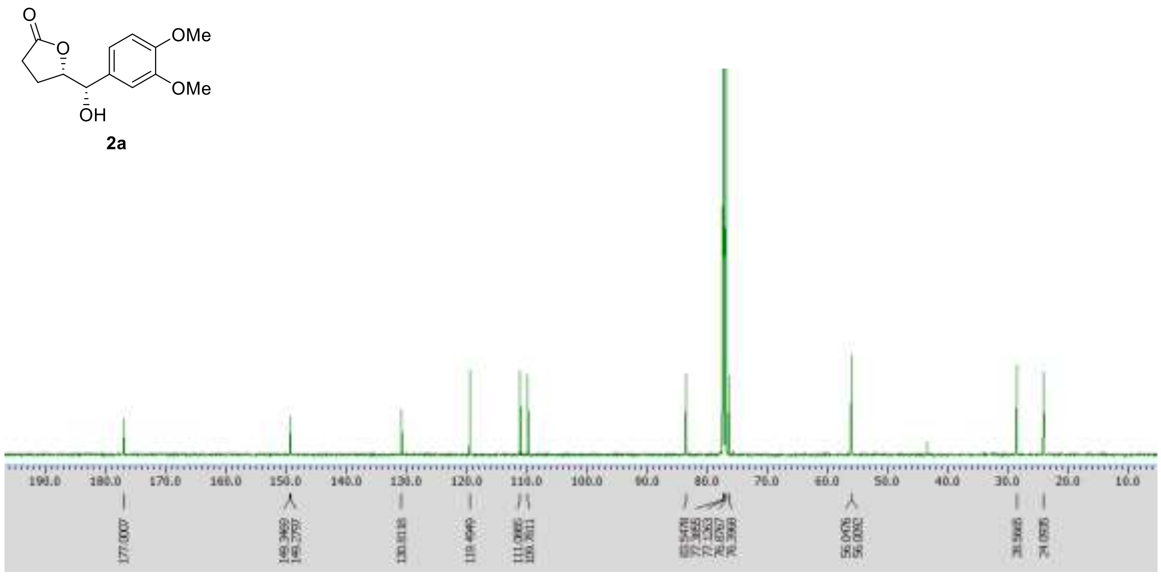

(*R*)-5-((*R*)-(3,4-dimethoxyphenyl)(hydroxy)methyl)dihydrofuran-2(3*H*)-one (**2b**)

[1]**H NMR** ($CDCl_3$, 500 MHz)

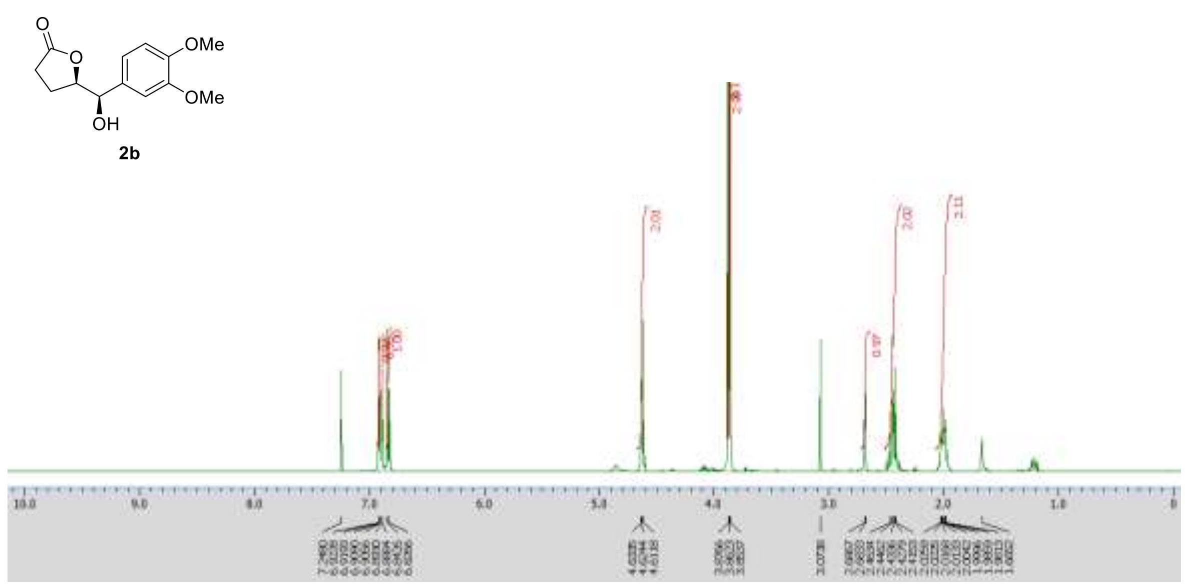

[13]**C NMR** ($CDCl_3$, 125 MHz)

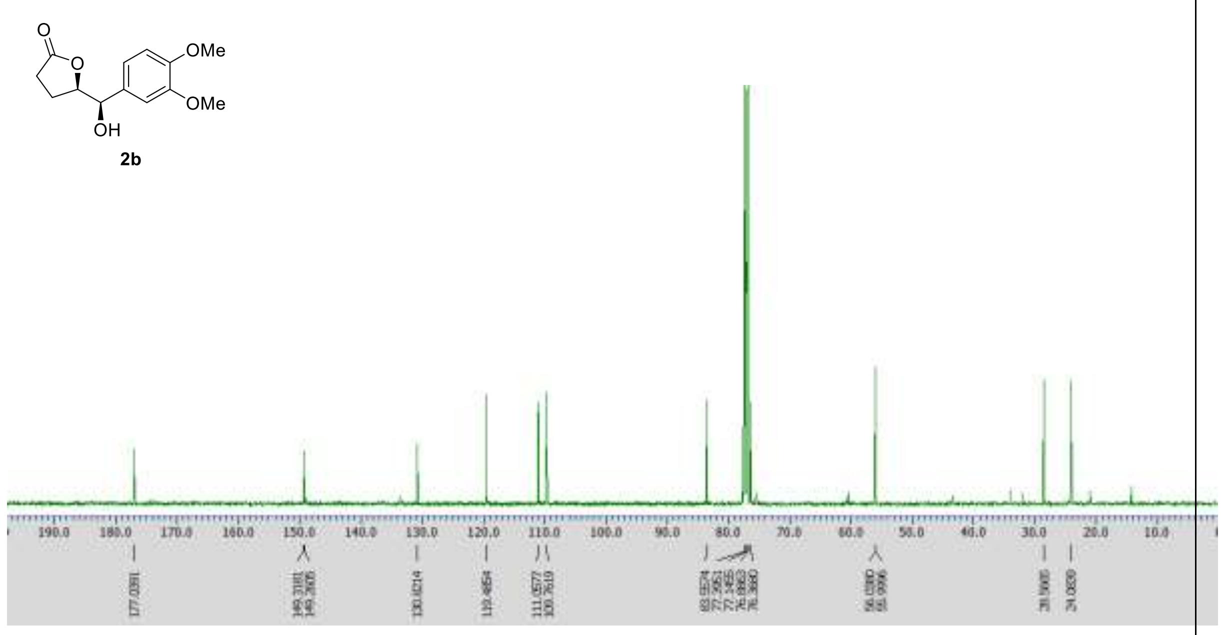

(*S*)-5-(3,4-dimethoxybenzyl)dihydrofuran-2(3*H*)-one (**7a**)

^{1}H NMR ($CDCl_3$, 500 MHz)

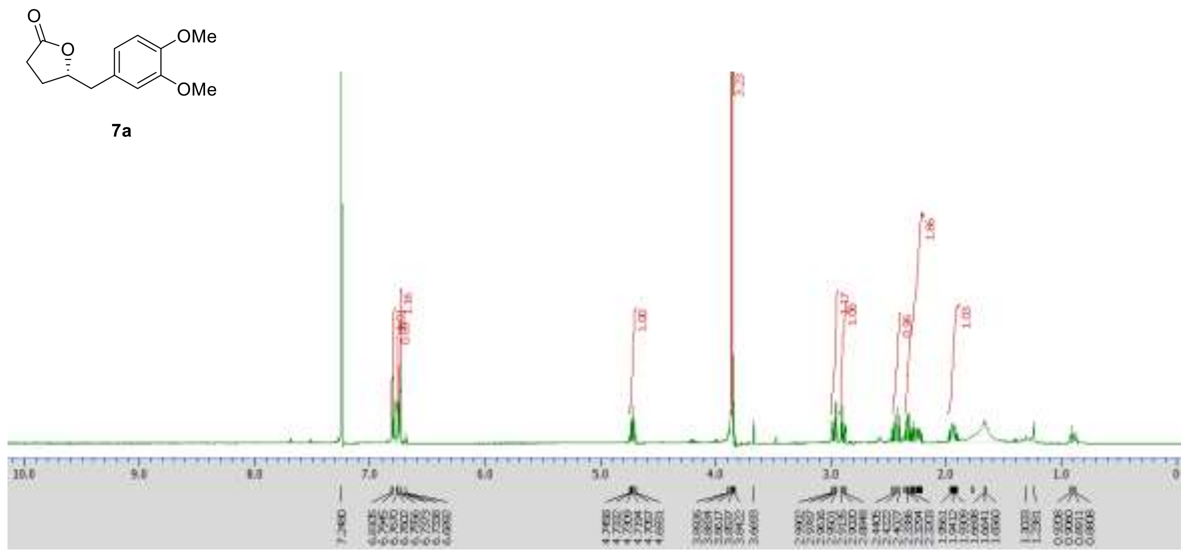

^{13}C NMR ($CDCl_3$, 125 MHz)

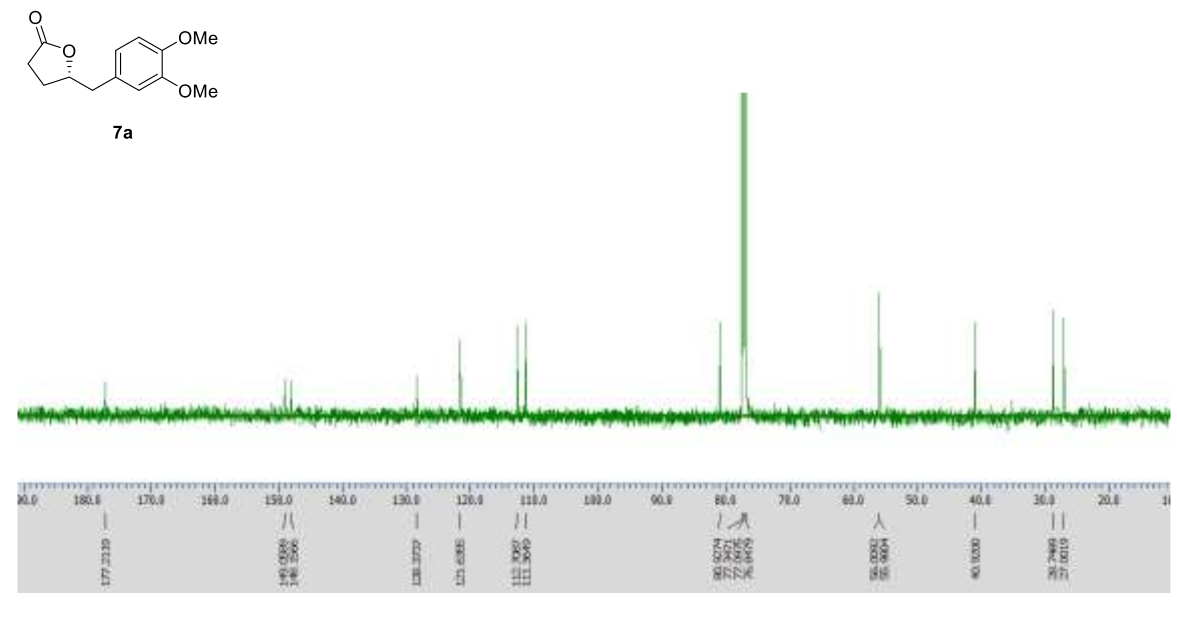

(*R*)-5-(3,4-dimethoxybenzyl)dihydrofuran-2(3*H*)-one (**7b**)

**^{1}H NMR** (CDCl$_3$, 500 MHz)

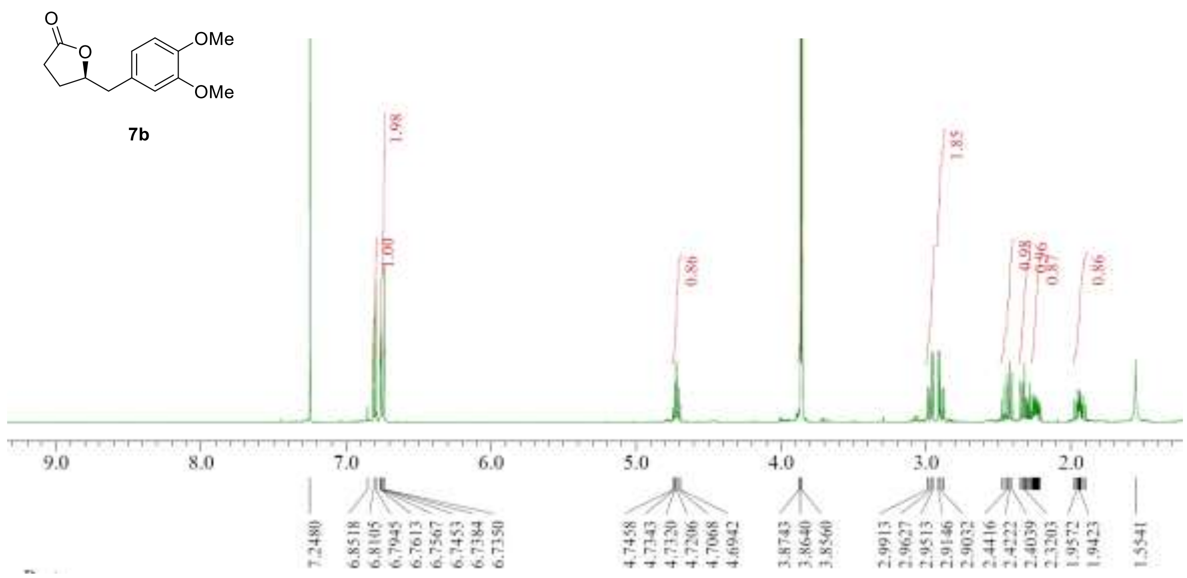

**^{13}C NMR** (CDCl$_3$, 125 MHz)

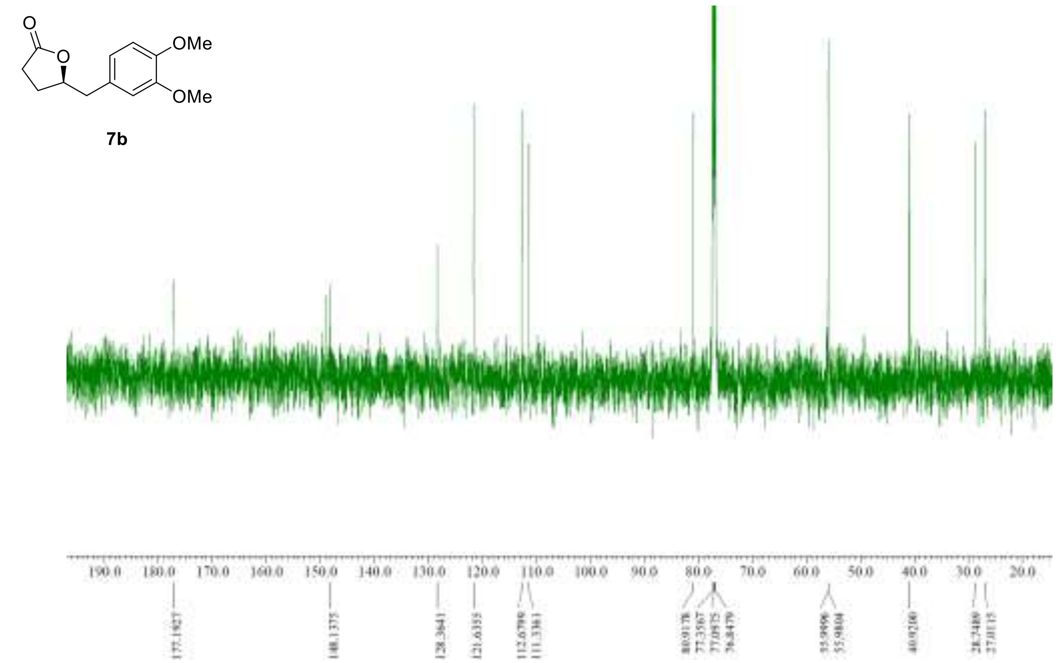

(*S*)-5-(3,4-dihydroxybenzyl)dihydrofuran-2(3*H*)-one (**1a**)

^{1}H NMR (DMSO-d_6, 500 MHz)

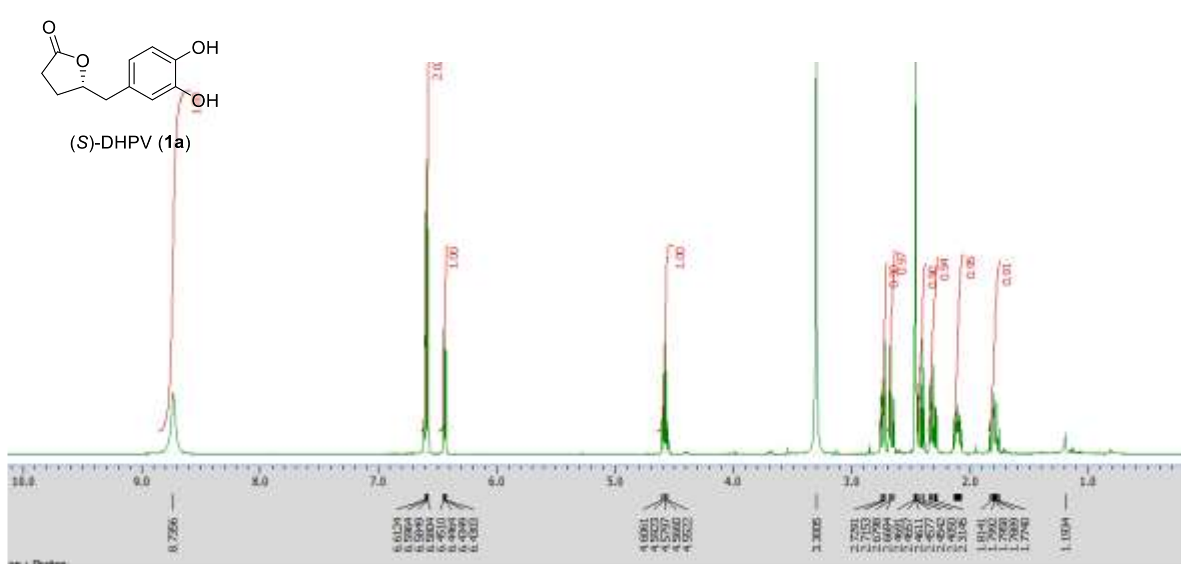

^{13}C NMR (DMSO-d_6, 125 MHz)

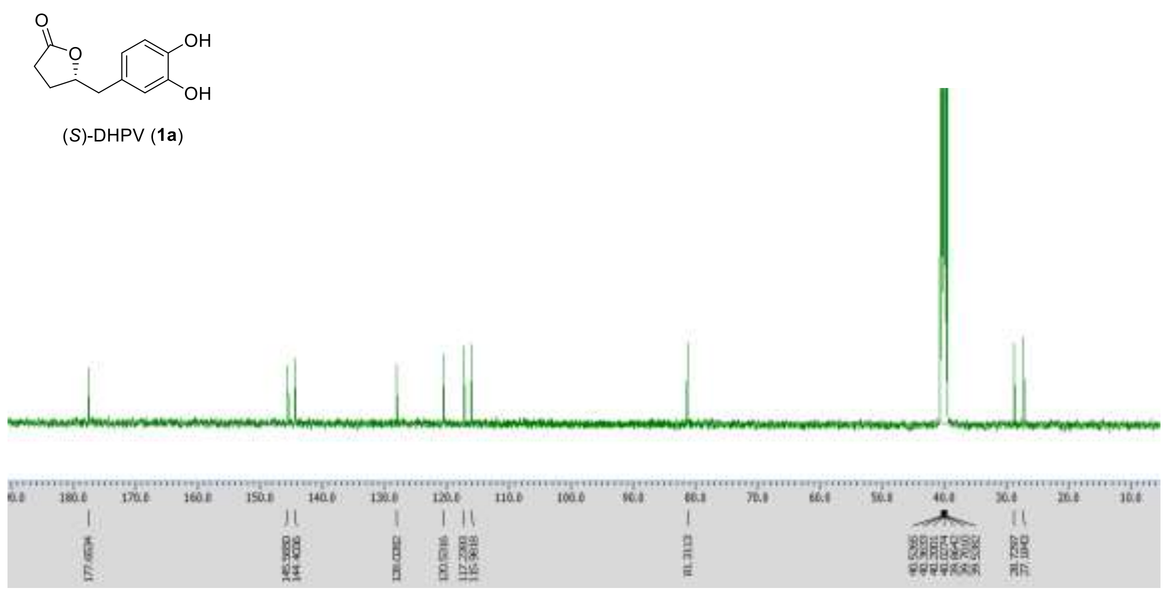

(*R*)-5-(3,4-dihydroxybenzyl)dihydrofuran-2(3*H*)-one (**1b**)

^{1}H NMR (DMSO-d_6, 500 MHz)

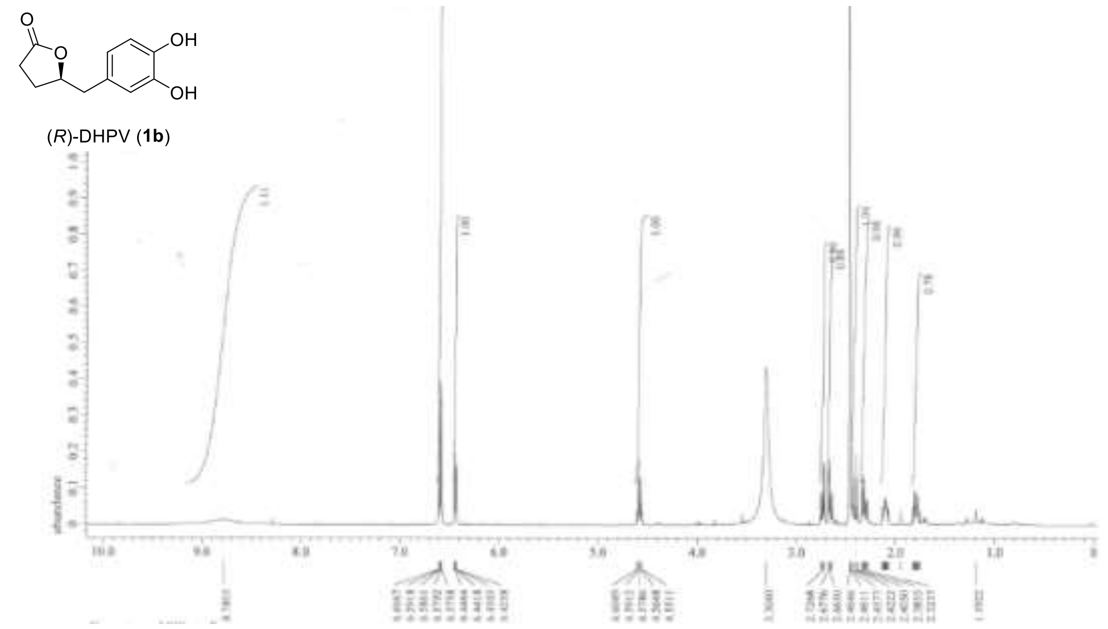

^{13}C NMR (DMSO-d_6, 125 MHz)

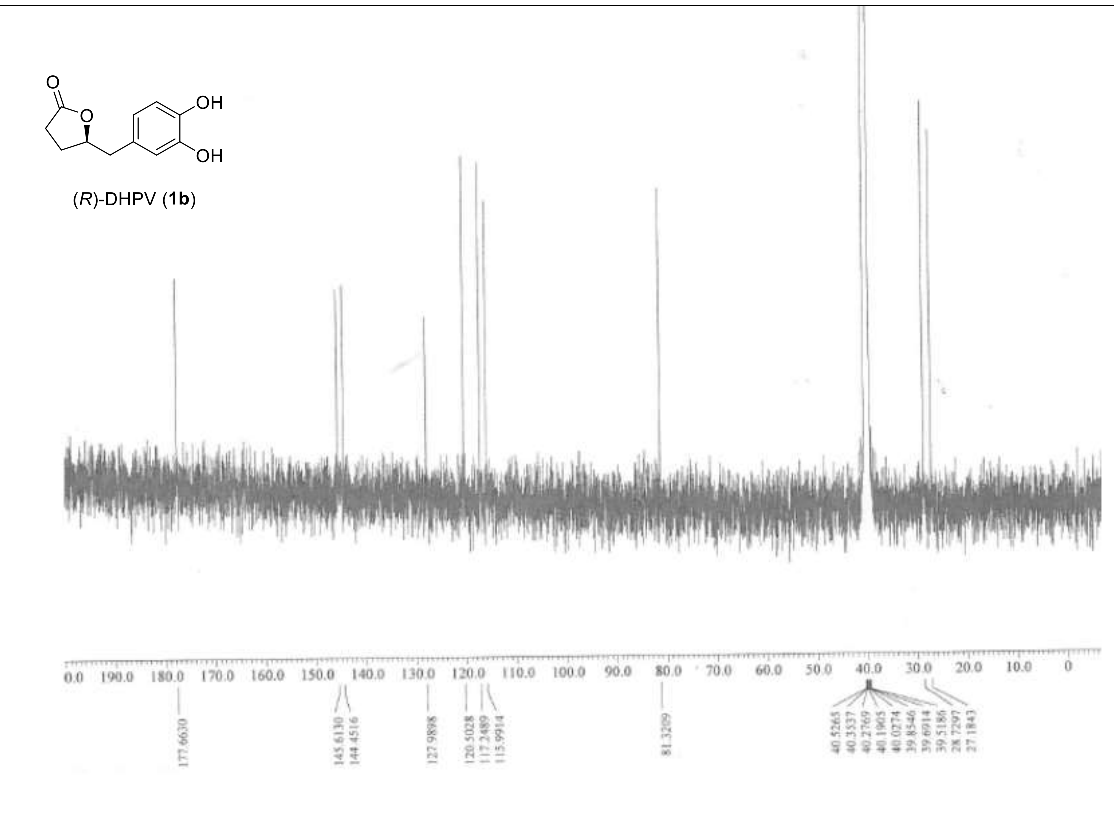

Table 1. Comparison of ¹H-NMR data for Synthetic and reported DHPV.

	(*R*)-DHPV	(*S*)-DHPV	(*R*)-DHPV	(*S*)-DHPV
	δ	δ	δ (mult, *J*)	δ (mult, *J*)
C2-CH_2	28.7	28.7	2.41 (dd, 17.8, 9.2),<br>2.31 (ddd, 17.2, 9.2, 4.6)	2.41 (dd, 17.8, 8.6),<br>2.31 (ddd, 17.8, 9.8, 5.2)
C3-CH_2	27.1	27.1	2.13-2.08 (m),<br>1.83-1.75 (m)	2.14-2.07 (m),<br>1.83-1.76 (m)
C4-CH	81.3	81.3	4.58 (quint, 6.7)	4.58 (quint, 6.7)
C5-CH2	40.2	40.2	2.73 (dd, 14.3, 6.8),<br>2.65 (dd, 14.3, 6.5)	2.73 (dd, 13.8, 6.9),<br>2.66 (dd, 13.7, 5.8)
C1’	127.9	128.0		
C2’-H	115.9	115.9	6.57 (d, 1.7)	6.58 (d, 2.3)
C3’-OH	145.6	145.5	8.78 (bs)	8.74 (bs)
C4’-OH	144.4	144.4	8.78 (bs)	8.74 (bs)
C5’-H	117.2	117.2	6.60 (d, 7.5),	6.60 (d, 8.0)
C6’-H	120.5	120.5	6.44 (dd, 8.0, 2.3)	6.44 (dd, 8.0, 2.3)

Chiral HPLC analysis of Synthetic compounds

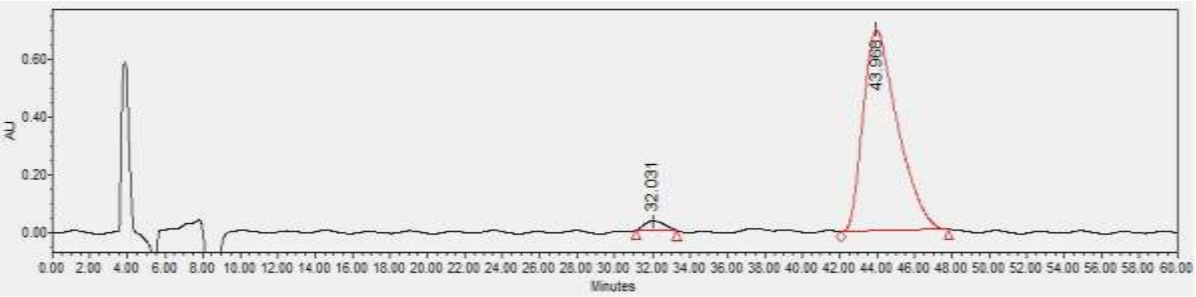

(*S*)-DHPV (**1a**)

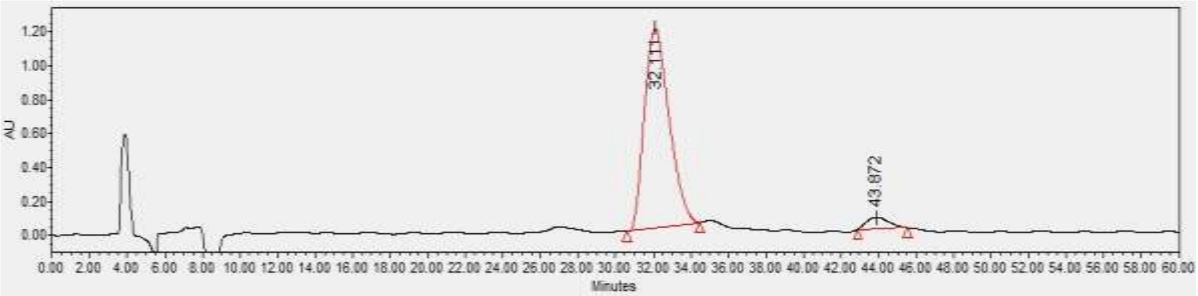

(*R*)-DHPV (**1b**)

Chiral HPLC chromatograms were obtained under the following conditions; Column, DAICEL CHIRALPAK® AD-H, 4.6 x 250 mm, 5 µm; detection, UV 210 nm; flow rate 0.8 mL/min; Mobile phase, A: Isopropyl alcohol, B: *n*-Hexane; Isocratic, A:B = 10:70

Figure S1. Chiral HPLC analysis of (*S*)-DHPV (**1a**) and (*R*)-DHPV (**1b**).
